# Absence of Red Blood Cell Alloimmunization in Transfused Patients Receiving Daratumumab: Experience from a Single Center

**DOI:** 10.3390/jcm14165754

**Published:** 2025-08-14

**Authors:** Lara Eritzpokhoff, Ernesto Talegón De La Fuente, Aida Carril Barcia, Pedro Asensi Cantó, Ines Gómez Segui, Mario Arnao Herraiz, Javier De La Rubia Comos, Pilar Solves Alcaina

**Affiliations:** 1 Blood Bank and Hematology Department, Hospital La Fe, 46026 Valencia, Spainsolves_pil@gva.es (P.S.A.); 2 CIBERONC, Instituto Carlos III, Fundación para la Investigación del Hospital Universitario y Politécnico La Fe de la Comunidad Valenciana, Hospital La Fe, 46026 Valencia, Spain

**Keywords:** alloimmunization, daratumumab, anti-CD38, interference, IAT, dithiothreitol, blood bank

## Abstract

**Background/Objectived:** Daratumumab is an anti-CD38 monoclonal antibody used in the treatment of multiple myeloma. Its use interferes with the indirect antiglobulin test (IAT). Treatment of reagent red blood cells (RBCs) with dithiothreitol (DTT) is one of the most validated techniques to resolve this interference. The objective of this study is to evaluate the rate of alloimmunization in transfused patients receiving daratumumab and the occurrence of hemolytic transfusion reactions. **Materials and Methods***:* We conducted a single-center, retrospective, descriptive analysis of all patients treated with daratumumab at our institution from October 2016 to April 2024. For daratumumab-treated patients requiring RBC transfusions, an IAT with DTT-pretreated RBCs (DTT-IAT) was performed using the automated Orthovision system. Transfusion was administered only with a previous negative DTT-IAT while respecting Rh and Kell phenotyping. We assessed the transfusion profile of our patient cohort, including their rates of alloimmunization before and after daratumumab initiation, as well as the incidence of hemolytic complications. Additionally, a literature review was performed on reported alloimmunization rates in daratumumab-treated patients. **Results:** Among all patients, 106 received RBC and/or platelet transfusions after starting daratumumab. Four had known pre-existing alloantibodies. None developed new alloantibodies or experienced hemolytic complications while receiving anti-CD38 therapy. There were four cases of false-positive DTT-IAT due to residual drug interference or technical variability, in which no alloantibodies or adverse transfusion reactions were detected. **Conclusions:** Patients receiving daratumumab exhibit a low risk of alloimmunization. This may be partly explained by adherence to Rh and Kell phenotyping and daratumumab’s immunosuppressive effects on alloantibody production. These results support the conclusion that an extended red blood cell phenotype or genotype before starting daratumumab could be omitted if a fast and reliable technique for pretransfusion testing (such as automated DTT-IAT) is available 24 h.

## 1. Introduction

Daratumumab, an IgG1-kappa monoclonal antibody targeting CD38, has become a pillar of multiple myeloma treatment, both in the newly diagnosed [1,2] and relapsed/refractory settings [3]. Apart from plasma cells, the CD38 transmembrane glycoprotein is also highly expressed on red blood cells (RBCs). For this reason, serum and plasma from patients under daratumumab induce pan-agglutination in the indirect antiglobulin test (IAT), leading to interferences in pretransfusion testing [4,5].

The anti-CD38 interference raises the concern that blood banks may miss potential alloantibodies during pretransfusion testing, which could lead to alloimmune acute and delayed hemolytic reactions with potentially severe consequences for the patients. Increasing evidence supports a similar or lower risk of alloimmunization as compared to general alloimmunization rates [6].

Different methods have been proposed to overcome these interferences. The use of Dithiothreitol (DTT), an enzymatic agent that destroys CD38 antigens on the RBC surface, is one of the validated techniques to eliminate anti-CD38 interferences in blood bank testing [7]. In fact, it is the most extended due to its simple realization and lower cost. The disadvantage is that the DTT treatment destroys not only CD38 but also other possibly relevant RBC antigens such as the Kell system. However, there is no standard practice globally and thus each bank can use different approaches depending on its capacity and resources.

In addition, the patient’s extended RBC phenotype or genotype is usually determined before starting daratumumab treatment [8].

The objective of this study was to retrospectively analyze the transfusion management of patients treated with daratumumab at La Fe Hospital, Valencia. The study reports the blood component transfusion burden before and after initiating daratumumab therapy and the RBC alloimmunization rate.

In addition, a review was carried out on all articles evaluating the alloimmunization rate in patients treated with daratumumab published until December 2024.

## 2. Patients and Methods

### 2.1. Patients

We retrospectively reviewed all patients treated with an anti-CD38 monoclonal antibody from October 2016 to April 2024 at La Fe Hospital in Valencia, Spain. All patients receiving daratumumab and with a diagnosis of multiple myeloma, AL amyloidosis, or plasma cell leukemia were included. Data to be collected were diagnosis, lines of treatment, previous autologous or allogeneic stem cell transplantation, the number of red blood cell and platelet transfusions before and after initiating the anti-CD38 therapy, the results of IAT before and after initiating anti-CD38 therapy, the rate of alloimmunization before and after initiating anti-CD38 therapy, and adverse effects of transfusion. The date and results of the last available IAT and the date of the last transfusion were collected. This study was approved by the Ethics Committee of La Fe Hospital, Valencia. The study was conducted in accordance with the Declaration of Helsinki and approved by the Institutional Review Board of Instituto de Investigación Sanitaria La Fe (2142_23, 20 December 2023) for studies involving humans. Since this was a retrospective study, in which there was no intervention, it was exempt from written informed consent.

### 2.2. Transfusion Management

Before starting anti-CD38 therapy, the ABO/Rh group, antibody screen, and extended RBC phenotype (including Cc/Ee, Kell, Fya/Fyb, JKa/JKb, and MNSs)—or genotype if the patient had been transfused in the last 3 months—were determined for all patients. When a transfusion was required once daratumumab had been started, the method used for the antibody screen was a manual IAT using a gel methodology with in-house DTT-pretreated commercial RBCs (DTT-IAT), as previously reported [9]. Our hospital introduced and validated a DTT technique since the first patients were treated with anti-CD38 in our center in 2016. In order to have access to DTT-pretreated RBCs at any time, especially in emergency situations, and to save time in pretransfusion testing, we used a preparation technique for commercial screening panel RBCs that prolongs their shelf life to 30 days [10]. In November 2022, we validated an automated gel methodology antibody screen (Orthovision, Ortho Clinical Diagnostics, San Diego, CA, USA) using DTT-treated RBCs (0.8% Surgiscreen, Ortho Clinical Diagnostics, San Diego, CA, USA). Patients receiving anti-CD38 were transfused if they had a negative pretransfusion DTT-IAT using electronic crossmatch, with Rh-compatible and K-negative RBCs. In case of a positive DTT-IAT, we carried out an identification using a panel of RBCs treated with DTT. When an alloantibody was detected, the RBCs selected for transfusion were negative for the corresponding antigen. Since 2024, the soluble CD38 protein (sCD38) from Grifols has been available in our blood bank to eliminate anti-CD38 interference in the plasma of patients with positive DTT-IAT. SCD38 adsorbs anti-CD38 antibodies in the patients’ plasma, thus eliminating the interference and allowing a standard IAT to be performed. This technique is easier and faster than DTT-IAT, but it is more expensive, and it is used only to solve cases of positive DTT-IAT.

### 2.3. Statistical Analysis

Computer software SPSS (version 15, SPSS Inc., Chicago, IL, USA) was used to perform the statistical analysis. Descriptive statistics are presented for variables. Results are expressed as median and range for continuous variables and as numbers with percentages for categorical variables. The Wilcoxon test was used to compare transfusion requirements between the related groups. *p* < 0.05 was considered significant.

### 2.4. Review of Literature Search Strategy

We first conducted a review of the literature about anti-CD38′s interference with pre- transfusion tests by introducing the words “daratumumab” and “alloimmunization”, “transfusion”, or “interference” into the Pubmed search system. We then selected only the articles that evaluated alloimmunization rates in patient cohorts treated with daratumumab, compiling all published data available until December 2024).

## 3. Results

### 3.1. Patients’s Characteristics

A total of 206 patients received treatment with daratumumab from October 2016 to April 2024 at our center. Patients’ characteristics are reported in Table 1. The principal indication for receiving daratumumab was multiple myeloma; however, we also included patients with other diagnoses. With a median follow-up of 1.33 years (0–4.83), 128 patients were still alive at the end of follow-up (62.1%).

### 3.2. Transfusion Requirements

Out of 206 patients, 148 patients (71.8%) received at least one RBC and/or platelet transfusion in their life until the last data collection (25 April 2024). Before initiating daratumumab, 100 patients received at least one RBC and/or platelet transfusion, and a total of 106 patients (58 of whom had been previously transfused) received at least one RBC and/or platelet transfusion after initiating daratumumab therapy. The median time between the first and last transfusion, once anti-CD38 had been started, was 0.7 months (0–53.2). The details of the transfusion requirements are summarized in Table 2.

### 3.3. Alloimmunization Rate

Out of all patients, only four (1.94%) had a positive IAT prior to starting daratumumab therapy because of previous alloimmunization, with the following specifics for each patient: one patient had anti-Fya, one had both anti-Wra and anti-A1, one patient had anti-c, and one had an unidentified alloantibody.

During follow-up, a total of 467 DTT-IATs were performed, with a median of 1 (range 0 to 32) per patient.

Only four DTT-IATs were positive, and none corresponded to a true new alloimmunization:

- One had an anti-c specificity in a patient that was previously alloimmunized (corresponding to an already known alloantibody).
- One was a false positive resolved with a negative non-automatized DTT-IAT.
- Two were false positives resolved with compatible crossmatch with selected RBC units treated with DTT.

Transfusions were well tolerated, and no hemolytic or any other adverse transfusion reactions were reported in patients transfused after having initiated daratumumab.

In 31 daratumumab-treated patients, a negative standard IAT was obtained after discontinuing daratumumab therapy. The median time between the last transfusion and daratumumab discontinuation was 335 days (76–1491). Only in three cases was the last transfusion performed before the end of daratumumab. The median time between the last transfusion during the daratumumab interference period and the last negative IAT was 196 days (9–4817). In another 18 patients, a negative DTT-IAT was confirmed at least one week after their last transfusion. Overall, in these 49 patients (46.2% of transfused daratumumab patients), we were able to confirm the absence of alloimmunization following their last transfusion. We could not confirm or rule out alloimmunization in the rest of our cohort due to lack of follow-up or death, although the last DTT-IAT available was negative in all patients.

### 3.4. Review of the Literature

We performed a review of the literature available on alloimmunization rates in daratumumab-treated patients that received transfusion from 2017 to 2024. By doing so, we update the review proposed by Bullock et al. [11] in 2021. The data of interest is summarized in Table 3.

## 4. Discussion

This study shows a low risk of alloimmunization in patients receiving daratumumab. Notably, this risk remained virtually zero despite the relatively high transfusion burden of our group. In addition, no hemolytic transfusion reactions were observed.

Patients diagnosed with multiple myeloma are among those with the lowest transfusion rates, as compared to other hematological malignancies such as acute leukemia [25]. Indeed, in different studies, the transfusion rate in daratumumab-treated patients ranges from 31% (n = 45/145 in Ye et al. [19]) to 59.4% (n = 145/244 in Tauscher et al. [20]). In our study, 44.6% of patients received RBC transfusion after initiating daratumumab, and there was a significant difference in transfusion rates before and after starting daratumumab therapy, both for RBCs only and when all transfusions considered (RBCs and/or platelets). This difference could be explained by the fact that daratumumab was initially used in refractory or relapsed myeloma, and these patients could have had a higher rate and severity of anemia. In addition, only a small percentage of multiple myeloma patients develop alloantibodies against red blood cell antigens, as shown by results of the present study (1.94% before starting anti-CD38) and others (see Table 3). Despite this fact, the possibility of alloimmunization after starting daratumumab is a concern for blood banks, since the interference of the monoclonal antibody in pretransfusion tests can put patients at risk of transfusion adverse events by making alloantibody identification difficult. The relatively low incidence of alloimmunization found in our study may be explained by three factors. Firstly, the immunoparesis induced by multiple myeloma itself, as previously described [26], might explain the low prevalence of alloimmunization before starting daratumumab found in our cohort and in previous reports. Immunoglobin levels or lymphocyte subsets were not analyzed in our study. Secondly, the cytotoxic effect of daratumumab on plasma cells may impair the capacity of patients to produce antibodies, including alloantibodies [27]. The final factor is the cautious transfusion policy established at our institution, which includes determining the extended phenotype/genotype of patients prior to daratumumab initiation and the delivery of Rh- and Kell-matched RBCs.

It should be noted that almost half of the patients receiving anti-CD38 therapy required transfusion in our study. In this sense, we consider that the lack of alloimmunization and any other adverse effects related to transfusion found in our patient population is a relevant contribution. Although there are other authors who also failed to detect RBC alloimmunization after anti-CD38 therapy, the numbers of patients reviewed were significantly lower than ours (Table 3). The largest study, analyzing 734 patients, found an alloimmunization rate of 2.86% before and 0.88% after starting anti-CD38 (3 of 341 patients transfused), respectively [11]. In most of the reviewed studies, the alloimmunization rate before anti-CD38 treatment was higher than that after therapy was started. Therefore, it seems that daratumumab-treated patients have a similar or lower alloimmunization rate than the standard patient population, ranging from 2 to 6%, according to Karafin et al. 2018 [28]. Only in the study reported by Cushing et al. [16] was the alloimmunization rate higher after starting anti-CD38 therapy. These authors reported three new alloantibodies in 3 patients out of 31 transfused after starting daratumumab therapy, the median number of RBC units transfused per patient being 8 (range 3–13). Given the low number of patients in their study, these results should be assessed with caution.

Our strategy of using an automated test to perform DTT-IAT allowed us to deliver blood components at any time and without any transfusion adverse event. The advantages of automation are the simplification of the technique and a reduction in inter-operator variability and in turnaround time, as well as a simplification of the procedure. We had to face just a few cases of false-positive DTT-IATs, which shows the limitations of the DTT technique and which can be explained by a residual drug interference or by technical variability. Fortunately, new reagents, such as sCD38, are available, eliminating the anti-CD38 interference in plasma before testing in a simple and fast way, and they should be reserved for positive DTT-IAT cases [29].

In general, transfusion management was simple, except for those cases in which the DTT-IAT was positive, which occurred in a minority of patients. Then, transfusion had to be delayed if possible and alloantibody identification or crossmatch with DTT-treated RBCs had to be performed. Therefore, our results support the safety of transfusions in this population, with no need for determining an extended phenotype or genotype before starting daratumumab if a fast technique (such as automatized DTT-IAT) is available for emergency situations. Despite the availability of DTT-IAT, our center also performed RBC phenotyping/genotyping as an aid to resolve immunohematological issues arising during anti-CD38 therapy and to provide phenotype-matched RBCs in cases of positive DTT-IAT with insufficient time for complete alloantibody identification. Based on our study results, RBC phenotyping/genotyping could be omitted, apart from Kell antigen matching and except for women of childbearing age and patients who develop RBC alloantibodies. In summary, transfusion management of patients receiving daratumumab in our center has become a standard and efficient procedure.

We are aware that the present study has some important limitations. It is a single-center study, which could make the generalization and applicability of the results difficult. We also faced a lack of follow-up for approximately half of our cohort because of patient death or because there were no IATs available more than one week after the last transfusion. Therefore, we do not know whether patients without follow-up could have become alloimmunized. Despite these shortcomings, this study provides valuable information on the transfusion management of patients undergoing anti-CD38 treatment in real life. Indeed, it is one of the largest cohorts until now evaluating the alloimmunization rate in daratumumab-treated patients.

In conclusion, our results support a low risk of alloimmunization in patients receiving daratumumab. In addition, if a fast and reliable technique for pretransfusion testing (such as automated DTT-IAT) is available 24 h, the determination of an extended red blood cell phenotype or genotype before starting daratumumab could be omitted. The results of this study need to be validated in prospective studies.

## Figures and Tables

**Table 1:** Patients’ characteristics.

Sex (%, n)	Female 51.45% (n = 106), male 48.5% (n = 100)
Age (median, {range})	70 {31–90}
Diagnosis (%, n)	Multiple myeloma 85.9% (n = 177) Primary AL amyloidosis 12.1% (n = 25) Plasmatic cell leukemia 1.9% (n = 4)
Previous lines of treatment (median, {range})	1 {0–5} 40.3% received daratumumab as first-line treatment (n = 83)
Previous stem cell transplantation (SCT) (%, n)	Autologous SCT 34.5% (n = 71); 28.2% (n = 20/71) received it after having initiated daratumumab. 29.6% received a second autologous SCT (n = 21/71). Allogeneic SCT 6.3% (n= 13); 46.2% (n = 6/13) received it after having initiated daratumumab
Number of daratumumab cycles (median, {range})	8 {1–87}

**Table 2:** Transfusion requirements.

	Before Initiating Daratumumab	After Initiating Daratumumab	*p*-Value
Patients receiving RBC and/or platelet transfusion (%, n)	48.5% (n = 100)	51.5% (n = 106)	0. 037
Patients receiving RBC transfusion (%, n) RBC units (median, range) by patient	40.7% (n = 84) 5 (1; 43)	44.6% (n = 92) 3 (1; 50)	0.003 0.927
Patients receiving platelet transfusion (%, n) Platelet units (median, range) by patient	28.1% (n = 58) 3.5 (1; 85)	30.1% (n = 62) 3.0 (1; 56)	0.231 0.631
Patients receiving both RBC and platelet transfusion	20.3% (n = 42)	23.3% (n = 48)	0.782

**Table 3:** Comparison of alloimmunization rates in different studies in patients treated with daratumumab.

Study	Number of Patients	Number of Patients with Positive IAT Before Receiving Anti CD38 Therapy (%)	Alloantibodies Detected Before Receiving Anti CD38 Therapy	Rate of Alloimmunization Once Anti CD38 Therapy Commenced (%)	New Alloantibodies Detected Post-Therapy	Extended Phenotype-Matched Blood (Y = Yes; Otherwise Specified)
Anani et al. 2017 [12]	62 in total (number of transfused patients not specified)	1.61% (n = 1/62)	Anti-Jka	0% (n = 0)	None	Not specified
Chari et al. 2018 [13]	14 patients transfused after receiving daratumumab	14.3% (n = 2)	anti-D, anti-E, anti-K, anti-Jkb, anti-Fya, anti-Fyb, anti-S, and anti-Knops	0% (n = 0)	None	Y
Deneys et al. 2018 [14]	14 selected patients that received daratumumab (11 transfused after receiving daratumumab)	0% (n = 0)	None	0% (n = 0)	None	Y
Bub et al. 2018 [15]	5 patients transfused after receiving Daratumumab	0% (n = 0)	None	0% (n = 0)	None	Y
Cushing et al. 2019 [16]	91 in total (31 transfused after receiving daratumumab)	6.59% (n = 6/91)	Not specified	9.68% (n = 3/31)	Anti-C, anti-S and anti-Cob	Kell
Carreño-Tarragona et al. 2019 [17]	30 in total (11 transfused after receiving daratumumab)	1.0% (n = 3/30)	Anti-D, anti-C, anti-c and anti-E	0% (n = 0)	None	Y
Arnao et al. 2019 [18]	44 patients transfused after receiving daratumumab	0% (n = 0)	None	0% (n = 0)	None	Rh Kell
Ye et al. 2020 [19]	145 in total (45 transfused with RBCs after receiving daratumumab)	0% (n = 0)	None	0% (n = 0)	None	In some cases, only Rh Kell matched; in others, extended phenotype matched, number not specified.
Tauscher et al. 2021 [20]	244 in total (145 transfused with RBCs after receiving daratumumab)	2.87% (n = 7/244)	Not specified	2.76% (n = 4/145)	Anti-E, anti-Fyb, anti-Jka and anti-S	Rh Kell
Bullock et al. 2021 [11]	734 patients treated with daratumumab (crossmatch RBC units were issued for 341 of them)	2.86% (n = 21/734)	Anti-D, anti-C, anti-E, anti-K, anti-Fya, anti-Jka, anti-Jkb, anti-G, anti-S, anti-Lua, anti-P1, others not determined	0.88% (n = 3/341)	anti-D, anti-E and anti-Fya	Rh Kell
Phou et al. 2021 [21]	90 in total (52 transfused after receiving daratumumab)	5.56% (n = 5/90)	Anti-E, anti-c, anti-Fya, anti-Jka, anti-S	0% (n = 0)	None	Rh Kell
Aung et al. 2022 [22]	29 in total (number of transfused patients not specified)	3.45% (n = 1/29)	Anti-C, anti-D	3.45% (n = 1/29)	Not identified	Not specified
Safić Stanić et al. 2024 [23]	68 (38 transfused before initiating daratumumab; 48 transfused after receiving daratumumab)	7.89% (n = 3/38)	Anti-E, anti-K, anti-C	0% (n = 0)	None	Y
Biswas et al. 2024 [24]	48 in total (41 patients transfused)	Not specified	Not specified	0% (n = 0)	None	Y (n = 35) or only RhD Kell (n = 6)

## Data Availability

Data is contained within the article. The dataset is available from the authors on request.

## Abbreviations

The following abbreviations are used in this manuscript:

IAT	Indirect antiglobulin test
RBC	Red blood cell
DTT	Dithiothreitol
DTT-IAT	IAT with DTT-pretreated RBCs
sCD38	Soluble CD38 protein
